# A new method to measure mechanics and dynamic assembly of branched actin networks

**DOI:** 10.1038/s41598-017-15638-5

**Published:** 2017-11-16

**Authors:** Pierre Bauër, Joseph Tavacoli, Thomas Pujol, Jessica Planade, Julien Heuvingh, Olivia du Roure

**Affiliations:** 1 ESPCI Paris, PSL Research University, CNRS, Université Pierre et Marie Curie, Université Paris Diderot, Physique et Mécanique des Milieux Hétérogénes, UMR 7636, Paris, 75005 France; 2 Present Address: Technical University of Denmark, Department of Energy of Conversion and Storage, Roskilde, Denmark; 3 Present Address: Centre for Genomic Regulation, The Barcelona Institute of Science and Technology, and Universitat Pompeu Fabra, Cell and Developmental Biology, Barcelona, Spain

## Abstract

We measured mechanical properties and dynamic assembly of actin networks with a new method based on magnetic microscopic cylinders. Dense actin networks are grown from the cylinders’ surfaces using the biochemical Arp2/3-machinery at play in the lamellipodium extension and other force-generating processes in the cell. Under a homogenous magnetic field the magnetic cylinders self-assemble into chains in which forces are attractive and depend on the intensity of the magnetic field. We show that these forces, from piconewtons to nanonewtons, are large enough to slow down the assembly of dense actin networks and controlled enough to access to their non linear mechanical responses. Deformations are measured with nanometer-resolution, well below the optical resolution. Self-assembly of the magnetic particles into chains simplifies experiments and allows for parallel measurements. The combination of accuracy and good throughput of measurements results in a method with high potential for cell and cytoskeleton mechanics. Using this method, we observed in particular a strong non linear mechanical behavior of dense branched actin networks at low forces that has not been reported previously.

## Introduction

The cell uses its cytoskeleton to resist deformation and integrate mechanical cues from its environment. The highly dynamic nature of the cytoskeleton enables the cell to change its shape in such a way that it produces displacement. Both the mechanics and the migration process in cells depend on a specific polymer meshwork formed of actin and its protein partners. Actin forms polar polymers assembling mostly at one end while disassembling at the other end^1^. In a cellular context, the polymerization is tightly regulated and polymerization occurs in a coordinated fashion. Filaments of polymerized actin are present in many locations of the cell with different organization and different set of associated proteins^2^. Of particular interest is the lamellipodium, a structure found at the leading edge of cells migrating on rigid substrate. At the membrane, proteins of the Wiskott-Aldrich Syndrome protein family, WASp, activate a protein complex called Arp2/3. This complex binds to existing actin filaments and provides a template for new actin monomers to polymerize. The localized polymerization of actin filaments in the direction of the membrane generates forces that push the membrane forward and allows the cell to migrate and to push on obstacles. The Arp2/3 protein complex has also been shown to nucleate actin networks in the cortex^3^ and to be present at the site of clathrin mediated endocytosis where it produces invagination and helps with vesicle internalization^4^. In these processes, the interactions between biochemistry, mechanics, architecture and force production are still far from being fully understood^2^.

The possibility to recreate *in vitro* Arp2/3-generated actin networks has pushed forward our understanding of these mechanisms^5–7^. By functionalizing a surface with activators and providing this surface with actin, Arp2/3 and a few regulatory proteins, a dense network of actin can be assembled. This has been done on *μm* sized colloids^7^ and patterned surfaces^8^ of up to tens of *μm* ^2^. Around beads, a dense homogenous actin shell grows from the surface and, in specific conditions, internal stresses develop up to the breakage of the shell into a *comet* developing from a side of the bead^9^. Few teams have been able to mechanically probe these structures by integrating them into force measurement experimental setups. In the first instance a colloid was glued onto a glass cantilever and the actin comet was held with a micropipette^10^. Another route is to grow an actin network on a functionalized Atomic Force Microscope (AFM) cantilever^11,12^. Recently, the reverse approach was used by deforming an actin network grown from functionalized patterned surface with an AFM^13^. With these techniques nanonewton forces can be exerted on growing actin networks and the growth speed can be monitored. Additionally a stress can be applied at a time scale shorter than growth and the resulting deformation can be monitored, giving access to mechanical properties of such networks. However, these techniques exhibit a low throughput due to the technical difficulties of integrating the biochemical reconstruction *inside* the mechanical probe device and the impossibility of parallel measurements. These experimental hurdles have limited their widespread use and also precluded systematic studies done by varying biochemical conditions.

**Figure 1:**
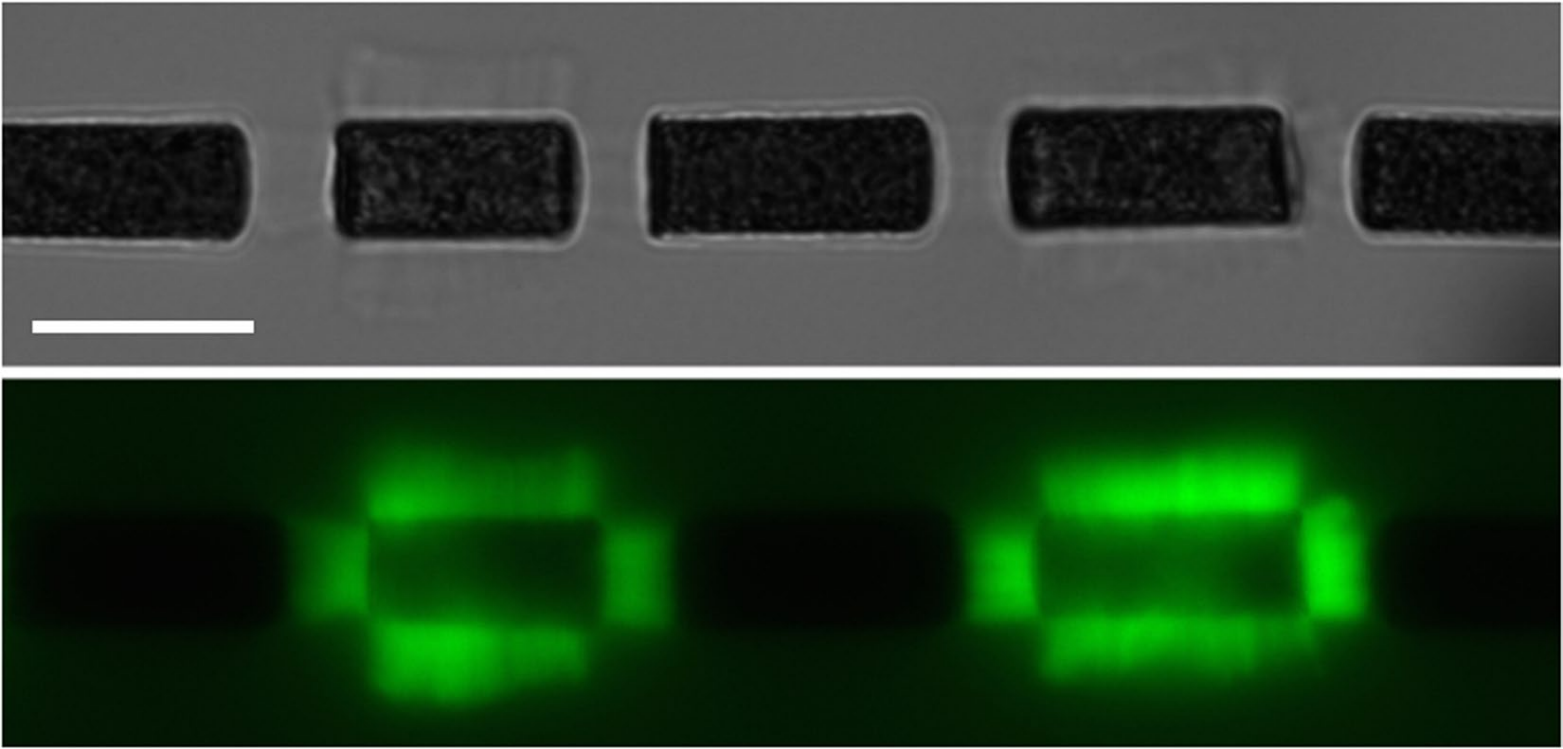
Chain of magnetic cylinders (6-*μ*m in diameter) in an horizontal magnetic field. Top: Bright field image, bottom, Fluorescent image (actin is labelled). Actin networks have grown from cylinders covered by the activator of the polymerization for around 15 minutes. Non actin-covered cylinders have been passivated by BSA. The cylinders are seen from the side, the circular faces being perpendicular to the image. Scale bar 10 *μm*.

Our approach consists of replacing the AFM setup by a pair of magnetic micron-sized-particles actuated - at a distance - by an homogenous magnetic field. In the presence of a magnetic field superparamagnetic particles self-assemble into chains in which forces are attractive and are controlled by the intensity of the magnetic field. These forces, from piconewtons to nanonewtons, are used to apply controlled stresses to the dense actin networks grown from the surface of one of the two particles - the other having been passivated - either to probe the mechanical properties or to follow the growth of the network as a function of an applied stress (see Fig. 1). This approach allows precise measurements to be carried out with a good throughput. The precision on the deformation - and thus on the mechanical properties - is achieved by measuring the displacement of the particles in bright field microscopy with nm-resolution, well below the optical resolution. The good throughput arises from the simple self-assembly of the magnetic particles into chains, and from the fact that several actin networks can be thus studied in parallel. We first developed this technique with spherical 4.5 *μ*m-particles from Dynal (Dynabeads M450 Epoxy)^14^. Commercial colloids have the advantage of being readily available, but the spherical geometry limits the number of accessible mechanical properties because of the non-linear relationship between force and indentation in a spherical contact described by the Hertz model^15^. Moreover, an actin comet cannot be handled and compressed between two spherical superparamagnetic colloids: as the particle can physically rotate while keeping the magnetic moment aligned with the field, the comet is pushed out from the chain so that the colloids get closer to each other which is more favorable energetically. To overcome these limitations, we developed the fabrication of anisotropic superparamagnetic colloids such as cylinders that cannot freely rotate as they prefer to align with the external magnetic field^16^. Importantly, with cylinders, the actin gel is compressed between two aligned flat surfaces, giving directly access to the stress - strain relation for mechanical probing.

The strength of this new method relies on three ingredients: 1) Applied stresses that are externally controlled and modulated, 2) Self-assembled structures which allow simple experiments and parallel measurements to be carried out and 3) Measurements of the relative displacements of the particles with nanometer resolution. This technique has been developed for the specific characterization of dense actin networks mechanics but can also be used to study other cytoskeleton meshworks, sub-cellular structures or even cells.

In the following, we will describe the different aspects of the method with detailed quantification of the performance as well as first results that can be obtained to understand mechanical and growth properties of Arp2/3-branched actin networks.

## Results

We will first describe the growth of Arp2/3-branched-actin networks from the surface of the micro-cylinders. We will then explain how we manage to perform accurate measurements of mechanical and growth properties of the networks. Finally we will give first results we obtained with this new method.

## Growth of actin networks from magnetic micro-particles

### Actin growth

**Figure 2:**
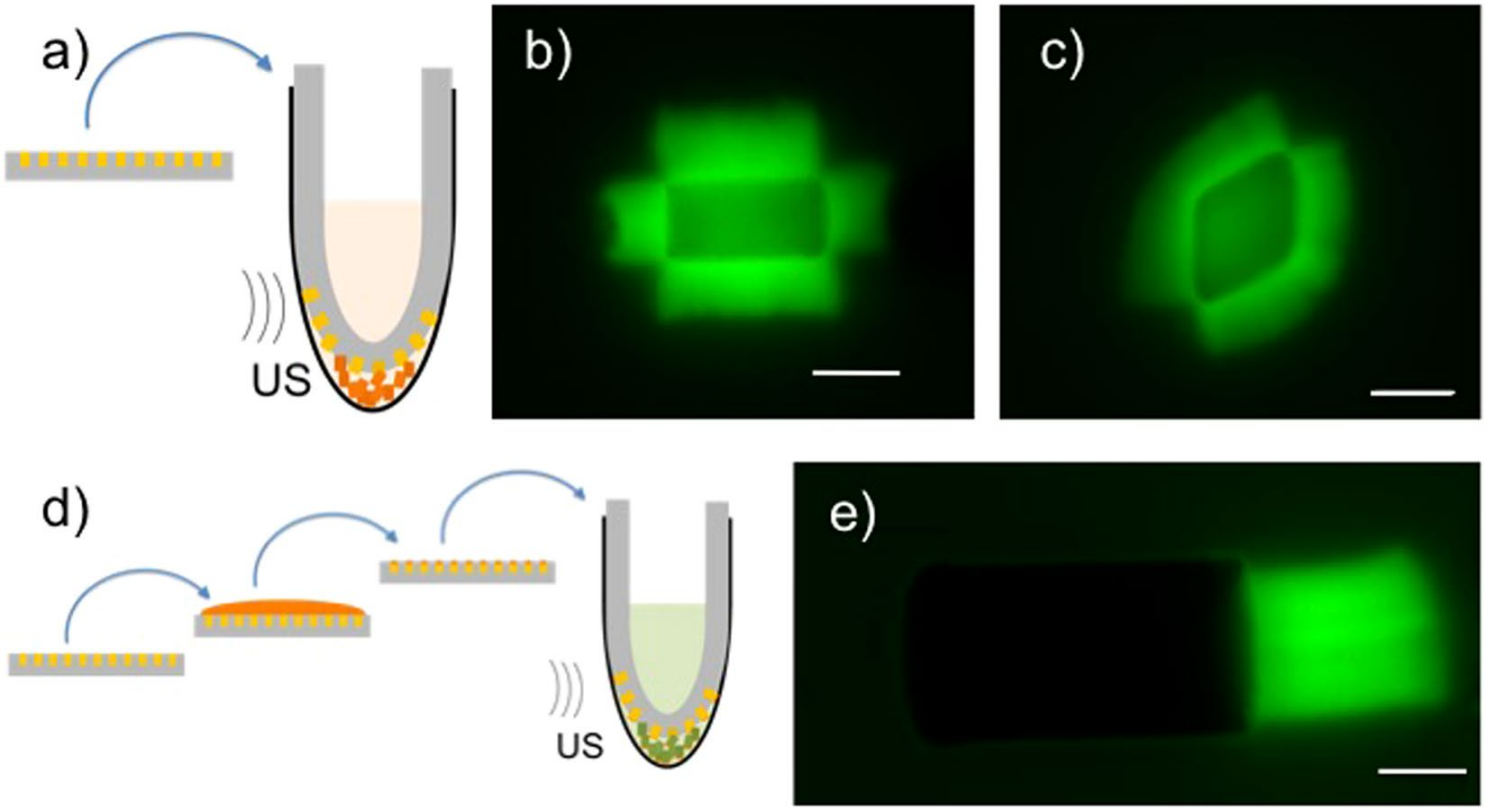
Extraction and functionalization of the cylinders. (**a**) and (**d**) are drawings explaining the principles. In both examples, as last step, the PDMS containing the cylinders is bent and introduced into an eppendorf tube containing a solution of protein which will graft quickly the cylinders as they come out from the mold. Ultra-sonic bath (US) helps the extraction from the mold. (**b**), (**c**) and (**e**) are fluorescent images of the actin networks grown from the cylinders. (**a**)–(**c**) 1-step extraction-grafting with VCA; (**b**) (**c**) fluorescent images of actin networks grown from a cylinder (**b**) and a diamond (**c**). Scale bars represents 6 *μm*. (**d**)–(**e**) 2-step grafting/extraction-passivation. One side-grafting by VCA is achieved by incubation for 30 minutes of a VCA drop (represented in orange) on the cylinders while still in the mold. Subsequently, the mold is bent into an Eppendorf tube and immersed in BSA solution for passivation (represented in green). This way we obtain 1-side functionalized cylinders as shown on figure (**e**). Scale bars indicate 4 *μm*.

Dense actin networks assemble from the surface of the cylinders as shown by the fluorescent images in Figs 1 and 2. Independent gels grow from the side and the two faces of the cylinder (see fluorescent images in Fig. 1). The two gels growing from the faces have a constant area and the force applied by the neighboring cylinder is homogeneously distributed on the gels’ surface. The applied stress can be directly calculated as the ratio of the force to the surface. In addition, actin growth from the two faces appears to be not limited by geometrical consideration. This is in contrast to the growth around beads where the gel growing from the surface is sterically limited by the preceding gel, leading to the build-up of internal stresses inside the network. The geometrical configuration of cylinders thus allows us to probe mechanically unconstrained actin branched networks.

### Fabrication and Functionalization of the cylinders

Briefly, the fabrication of the magnetic micro-cylinders^16^ relies on the filling of micro-sized PDMS-molds (made by standard soft lithography techniques) by a curable mixture of monomer and superparamagnetic small colloids (300 nm in diameter, Ademtech). As compared to our first implementation of the micro-fabrication published in Tavacoli *et al*.^16^, we use here a thermo-initiator (Azobisisobutyronitrile from Sigma) instead of a photo-initiator to improve reproducibility. We finally obtain small colloids embedded in a rigid matrix whose micron-sized shape is similar to the shape of the mold.

The first objective with this lab-made fabrication is to obtain particles that are magnetic enough: the careful choice of colloid surface chemistry and monomer allows the volume fraction of small colloids in our particles to be close to maximum, giving access to forces from piconewtons to nanonewtons. The second difficulty is the harvesting of the micro-cylinders from the mold and their bio-functionalization. The use of a sacrificial tacky layer of polymer as described in Tavacoli *et al*.^16^ proving non suitable, we developed a protocol which allows efficient harvesting and functionalization of the micro-cylinders at the same time to be performed. This strategy avoids micro-cylinders which are very sticky right after extraction from the mold either to aggregate or to stick to the tube. Practically, we introduce the PDMS layer containing cylinders bent into a small tube containing the protein to be grafted on and sonicate the tube to help the extraction (see Fig. 2). Cylinders are thus quickly covered either by the activator of the actin assembly (in the present case, the C-terminal region of WASp, called VCA fused with a tag Glutathione S-transferase called GST) or by Bovine Serum Albumine (BSA) for passivation. Alternatively, by incubating the protein on top of the PDMS layer, we managed to functionalize only one face of the cylinders. The remaning sides can then be passsivated or coated with a different protein as before (see Fig. 2c–e).

## Forces and distances

To measure mechanical properties or to follow the assembly of the networks when facing an opposing force (to mimic the polymerization of actin networks close to a membrane either in the lamellipodium or during endocytosis), one would need to apply controlled stress (or strain) and to precisely measure the resulting strain (or stress). In this section, we will show how we designed our experimental technique to have good control on both.

### Spatial resolution

#### Sub-pixel correlation allows nanometer precision on cylinders’ displacement

**Figure 3:**
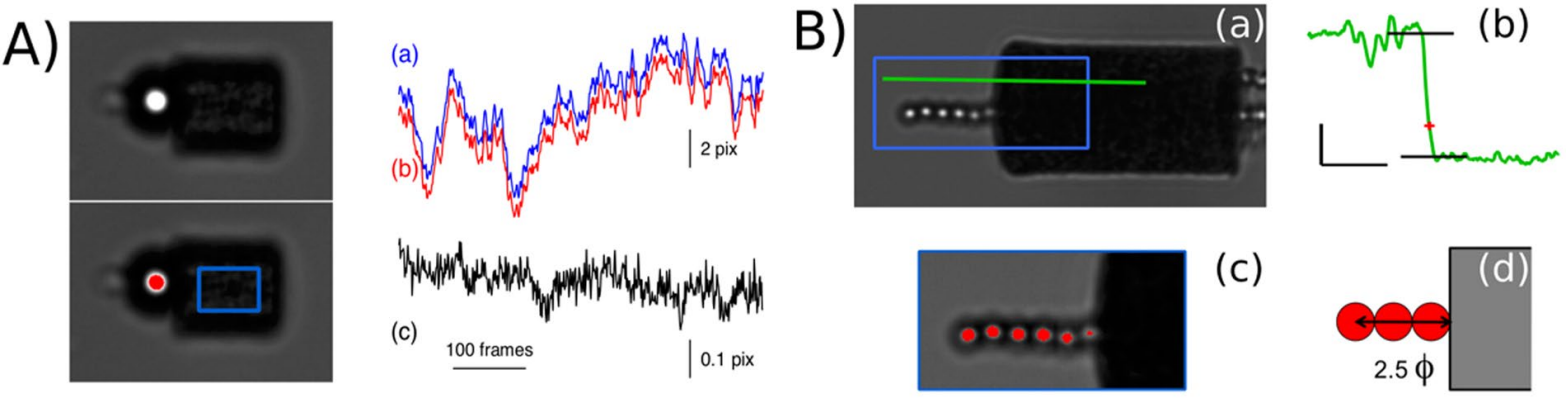
Spatial resolution. (**A**) A magnetic bead (4.5 *μ*m diameter) and a magnetic cylinder (6 *μ*m diameter) in dipolar interaction in a horizontal magnetic field of 20 mT. The white spot at the center of the bead (in red on the bottom image) is used to determine its position. The blue square is the region of interest used for image correlation on the cylinder (4 × 2.5 *μm* ^2^). Position of the cylinder measured with image correlation (a) and of the bead measured with the center of mass of the center spot (b), while enduring brownian fluctuations (x-axis 30 frames/s). The position of the bead is shifted by + 1 *μ*m for better comparison. (c) Difference in the position determination of the bead and the cylinder with the two methods (center determination and image correlation, equivalent to the difference from the red and blue curve from (a) and (b)). Note that the scale is much smaller for the difference (c) (1/20). The mean difference is less than 1/20th of a pixel corresponding to 2.5 nm and giving an upper bound to the resolution of the two independent methods of position measurements. (**B**) (a) A magnetic cylinder (8 *μ*m diameter) and several magnetic beads (Dynabeads my-one, 1.05 *μ*m diameter) in dipolar interaction; the rectangle square is the limit of the zoomed part on (c), whose dimensions are 13 × 5.5 *μm* ^2^. (b) A rough estimation of the position of the cylinder’s vertical surface is done by a threshold on grey level contrast measured on the green line of image (a). (c) and (d) Calibration of the estimation by using the chain of beads on the left part of the image whose position can be accurately determined. ${\varphi }$ is the diameter of the small beads.

The position of the center of spherical colloids can be determined accurately from bright-field images by calculating the center of mass of grey levels in the central spot of the diffusion pattern of the beads (Fig. 3). The displacement is then simply computed from the difference between position on two consecutive images. For cylinders, the displacement is determined by using sub-pixel correlation^17^ on a region of interest drawn inside a cylinder (represented by the blue rectangle on Fig. 3A). The accuracy of the procedure allows nanometer-resolution to be obtained and proceeds from the strong contrast of the images with dark and white spots, the large area they span, several 100 pixels^2^ with 100x magnification and the large dynamics of our camera (16bits, ORCA-Flash4.0 HAMAMATSU). By following a bead-cylinder pair submitted to Brownian fluctuations and hold together by a magnetic field we have been able to estimate the error on the displacement of both micro-objects to be $\le 2.5$ nm (see Fig. 3, more details will be found in Methods section).

#### Accurate measurements of the gel thickness

The thickness of the probed gels can be measured at low magnetic field through the distance between the two neighboring objects’ surfaces. The very low polydispersity in diameter of M450 Dynabeads ($< \mathrm{0.5 \% }$ ^14^) and the good resolution on the position of their centers give an accuracy of 20 nm on the thickness measurements. The determination of the position of the cylinder surface is not as straightforward as image correlation only provides a relative displacement of the micro-cylinder and no absolute position. We settled this problem by using simple image analysis (illustrated on Fig. 3B-a) and a calibration with small spherical magnetic colloids to increase the precision (see details in Method section and illustration in Fig. 3). The error on the length of the actin gels is evaluated to be 0.178 *μ*m. This is well over our relative displacement precision, but still below the optical resolution.

### Dipolar magnetic forces

**Figure 4:**
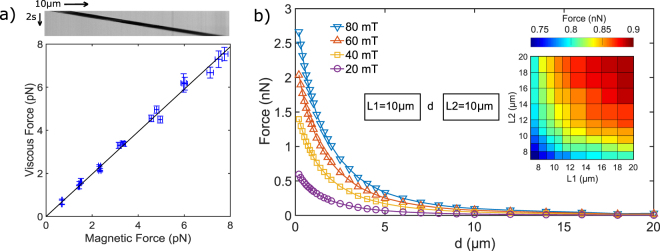
(**a**) Magnetic characterization. Comparison between viscous forces calculated from velocity measured on kymograph like the one on top and magnetic forces calculated from field gradient, field intensity, volume fraction of colloids in the solution used to fabricate the cylinders and the susceptibility of the small colloids. This magnetic response is used to calculate the magnetic forces between cylinders (see **b**). (**b**) Magnetic forces between cylinders calculated by finite elements analysis. The curves show the magnetic forces between two identical cylinders (10 *μ*m-long and 6 *μ*m in diameter) as a function of distance, d and for increasing magnetic field. Force decreases when cylinders separate and when magnetic field decreases. Inset, dependence of forces to the lengths of the cylinders, L1 and L2, calculated at d = 1 *μ*m and 40 mT.

For spherical colloids one can calculate the dipolar forces within the chain by the approximation of point like object; the force depends on the 4th power of distance between center of colloids, the magnetic properties of the beads and the square of the magnetic moment. For cylinders, we used finite element analysis to calculate the force between two cylinders using magnetic properties characterized by following the drift on the cylinders in a field gradient (see Methods). A parametric sweep was done varying the dimensions of the cylinders (diameter and length), the magnitude of the excitation field and the distance between the two surfaces (see Fig. 4). The calculated forces were then used to determine the force as a function of distance and excitation field for each pair of specific cylinders used in our experiment. The force reaches 2 nanoNewtons at 1 *μm* distance at the maximum excitation field of our setup ($6.4$ ${10}^{4}\,A/m$ corresponding to 80 mT). Forces decrease strongly with the distance between the cylinders and increase with magnetic field magnitude until saturation that corresponds to saturation of the magnetic response of the 300-nm colloids. At short distances, the forces depend only slightly on the lengths of the two cylinders: doubling the lengths induces an increase of the force around 16.5% for a distance between the cylinder faces of 1 *μ*m. The diameter of the cylinders has a strong impact on the force but a much smaller impact on the stress (i.e. force divided by the surface of the cylinder face) as shown in supplementary Figure 1. The variability in the magnetization of the cylinders is the main source of error in the force calculation. From the characterization of cylinders magnetic properties, we estimated a precision on the force of 8.6%.

### Cylinder’s alignment

Within the chain, a restoring force maintains the cylinders aligned on the same axis (see Fig. 1). Calculations with finite element analysis were performed for two misaligned cylinders of 6 *μ*m in diameter and 12 *μ*m in length subject to an external field of 40 mT. The restoring force towards the central axis is of the order of 100 pN for a cylinder shifted by 1 *μ*m out of the central axis. This value is large as compared to brownian forces and thus explains the very good alignement of the cylinders in the chains (See supplementary Figure 2). This alignement is crucial because it ensures real compression of the gel between parallel surfaces. The applied stress can easily been calculated as the ratio of the force to the area of the cylinders’ face.

As a conclusion, we are able to fabricate magnetic cylinders on which Arp2/3 actin networks can assemble without generation of internal stresses. The cylinders interact together in an homogenous magnetic field to form chain in which each cylinder attracts its neighbors with forces up to nanoNewtons. They allow real compression of actin gels to be applied with a very good control on the stress. In other words, we have developed and validated the tools necessary to study the growth and the mechanics of Arp2/3 actin networks with a large statistics.

## Mechanics and Growth of Arp2/3 actin networks

By developing this new experimental technique our goal was to study mechanical properties and dynamic assembly of Arp2/3 actin networks. More precisely we aim at understanding how the growth speed of the actin network is influenced by an external load as well as how they deform when submitted to mechanical stresses. In this last part of the results section, we present our results on these two questions.

### Dynamic assembly of Arp2/3 actin network

**Figure 5:**
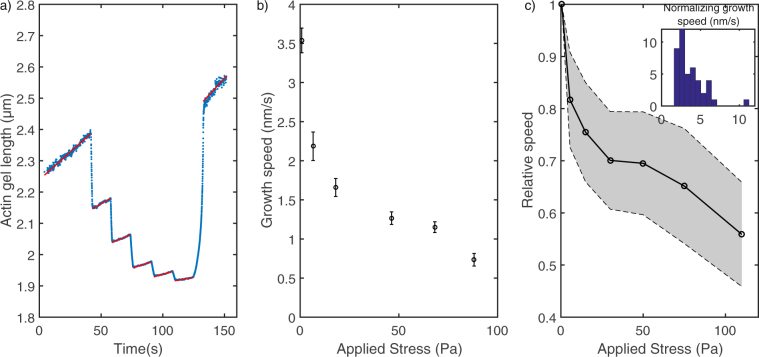
Stress-dependent growth. (**a**) Actin gel length measured by the distance between the faces of two cylinders as a function of time. The magnetic field is increased by 15 sec-steps from 3 mT to 80 mT. The displacement as a function of time is fitted at each step of constant magnetic field (red lines). (**b**) Growth speed as a function of applied stress for each step of the preceding curve. The applied stress is mostly constant at each step as the displacement of the cylinders is small. (**c**) Normalized speed as a function of applied stress for several actin networks, inset: distribution of the normalizing velocity measured at low stress. The shaded area represents the 95 % confidence interval.

As the actin network grows, the adjoining cylinders move apart from each other allowing the growth speed to be measured with an accuracy only limited by the nm range precision on the cylinders displacements. In order to investigate the dependance of this growth on an applied stress, we increased step by step the magnetic field in the experiment chamber. Constant field was maintained for 15 sec on each of the five steps and then relaxed to the original small field. Figure 5a shows the variation of the actin gel length as a function of time during these steps. Increasing the stress, which is directly the magnetic force divided by the area of the gel, results in reducing the growth speed as shown by the decreasing slopes of length-vs-time curves during the steps (fitted by a red line on Fig. 5a and reported as a function of stress on Fig. 5b). The same stepped increase of stresses was reproduced several times on the same network and no history dependent behavior was observed (see supplementary Figure 3 and 4). Figure 5c assembles multiple ($n > 30$) stepped increases of the stress on different networks grown with the same protein concentrations to show an average behavior. The observed relationship between growth speed of reconstituted actin branched network and the applied stress is in accordance with results at low applied stress from previous teams^10,13^.

Between the steps, an elastic compression occurs which can be used to measure the non linear mechanics of probed networks (see next section). When the stress is relaxed after the five steps, the network relaxes, showing minimal residual deformation. Indeed the length released by the actin gel after stress relaxation is within 5% of the sum of deformation at each compression step. Conversely, the length after the end of the compression cycle is compatible with the integration of the stress-dependent growth speeds during the time of the cycle.

### Mechanics of Arp2/3 actin networks

**Figure 6:**
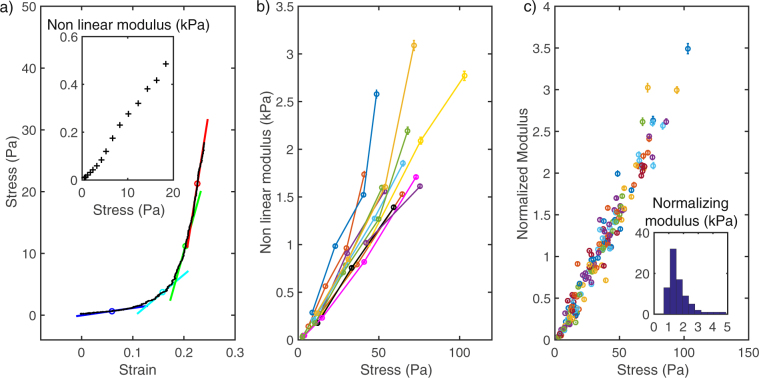
Non linear mechanics. (**a**) Stress-strain curve for a given actin network obtained by application of an increasing force. The tangent of the curve on four points is represented by short colored lines whose slope is a measure of the local non linear modulus. Insert: the non linear modulus measured all along the stress strain curve. (**b**) Dependence of the non linear modulus to the stress for 9 different gels. For each gel the dependence of non linear modulus to stress is linear allowing a modulus at 50 Pa to be extracted from a linear fit and used to compare gels. (**c**) Normalized non linear modulus as a function of the stress, normalization is done with the modulus at 50 Pa.

The elastic modulus quantifies the response to a strain or a stress of a material. Here we are probing the mechanical response of actin networks by applying a given stress and measuring the induced strain (deformation normalized by the undeformed length). For usual elastic materials the stress/strain curve is linear and the elastic modulus is directly the slope of this curve. On Fig. 6a is shown the stress-strain curve for an actin gel submitted to a stress increasing from 0 to 30 Pa. One can easily see that the curve is not linear meaning that the elastic response depends on the applied stress: the larger the stress, the stiffer the gel. Such non-linear mechanics has already been reported for actin suspensions and gels^12,18^. It can be quantified by measuring the local slope of the stress-strain curve (see Fig. 6a). On the inset, the local elastic modulus - also called non linear modulus - is plotted as a function of the stress.

Another way to measure the dependence of elastic modulus on the stress is to measure the deformation in response to a stress increment around a given value σ, as mentioned in the previous paragraph. The non linear modulus can be measured as the local elastic modulus, ${\rm{\sigma }}/\varepsilon$, where $\varepsilon$ is the induced strain. This procedure has been used to compare 20 different gels grown with similar biochemical conditions in the experiment summarized in Fig. 6b where we plotted the non linear modulus as a function of σ.

Given the very reproducible linear shape of the non-linear modulus dependence on the stress, we use the slope of this curve to quantify the elasticity of the network. This slope is measured with a fit using the modulus measured at the different stresses. As this slope is non-dimensional (Pa/Pa), we prefer to quantify the non linear behavior through the non-linear modulus at a given stress (calculated by the product of the slope and the given stress) and we chose 50 Pa because it sits nicely in the upper range of our forces. Figure 6c is the normalized plot showing the large reproducibility obtained when comparing the stress-stiffening behavior of different gels. The inset shows the distribution of the modulus at 50 Pa. We expect this distribution to depend on the architecture of the gels and thus on the composition of the growth medium^14^.

## Methods

### Growth of actin network on magnetic micro-cylinders

#### Fabrication and functionalization of magnetic cylinders

Briefly, PDMS molds are prepared by soft lithography and treated with fluoro-silane vapor deposition. Cleaned and dried silica superparamagnetic beads (Ademtech) are mixed with ETPTA (Ethoxylated TrimethylolPropaneTriAcrylate from Sigma)+3.5% v/v Azobisisobutyronitrile (Sigma). The dispersion is then poured into the PDMS wells by gently spreading the solution above it. Reticulation was ensured by placing the filled PDMS molds at 135 °C for 2 hours. To extract the cylinders, a strip of filled PDMS mold (typically 40 mm length, 3 mm wide and 2 mm depth) is bent and introduced into a 0.5 *μl* eppendorf tube containing the solution of protein to be grafted (see Fig. 2). The eppendorf tube is then put in an ultra-sound bath at 38 kHz and 25 W for 5–30 minutes. Cylinders are mostly extracted in a thin band at the most curved part of the strip, which can be checked by the bleaching of this band. The time of extraction depends mainly on the quality of silane treatment and the aspect ratio of the cylinders (longer times are needed for longer cylinders). The extraction solution was either VCA-GST at 0.64 *μM* or BSA at 10 mg/ml.

As the wells are filled by a wetting mechanism^19^, the upper face of the cylinder exhibits a small negative curvature which is a signature of the meniscus. No differences in the growth of actin networks on the bottom or top (slightly curved) surface were observed (actin length of $1.56\pm 0.17\,\mu m$ after 10 min polymerization from top surfaces, compared to $1.75\pm 0.34\,\mu m$ from bottom surfaces).

#### Composition of growth medium

The proteins used here are kind gift from Marie-France Carlier’s lab in Gif-sur-Yvette (LEBS, France). The composition of the actin network growth medium was alpha-skeletal actin purified from muscle actin (6.5 *μ*M), Gesolin (90 nM), Arp2/3 (180 nM), and ADF (6.5 *μ*M) in a buffer of Hepes 6 mM, ATP 1.8 mM, DTT 6.2 mM, Dabco 140 *μM*, KCl 80 mM, CaCl_2_ 100 *μM*, MgCl_2_ 6 mM at pH 7.8. Actin was kept in G-buffer (5 mM Tris HCl pH7.8, 200 *μM* CaCl_2_, 200 *μM* ATP, 1 mM DTT) and polymerized prior to its mixing with the growth medium byby adding 1/20 of final volume of KME (KCl 2 M, MgCl_2_ 20 mM, EGTA 4 mM).

### Force-distance measurements

#### Set-up: microscope and field generation

The setup is mounted onto a Zeiss inverted microscope (Axio A1 Carl Zeiss, Germany) equipped with X-cite XPS-120 lamp for fluorescence. Observations are made through an oil-immersion 100x objective (NA = 1.4) and recorded with a Orca4 sCMOS camera (Hamamatsu Photonics, Japan). To generate homogenous magnetic field, the stage has been modified to hold two coaxial coils (length 40 mm, diameter: inner, 26 mm, outer, 88 mm, 750 spires, SBEA, Vitry, Fr) with mu metal core, their axes being in the focal plane. The coils are powered by a bipolar operational power supply amplifier 6 A/36 V (Kepco, Flushing, NY) controlled by Labview software (National Instruments, Austin, TX). The maximum field generated is 80 mT with a gradient less than 0.1 $mT\mathrm{.}m{m}^{-1}$ over the sample.

#### Sub-pixel correlation: determination of cylinders’ displacement

A sub-image is selected inside the image of a cylinder, typically spanning 120 × 70 pixels. The correlation is calculated between the reference sub-image and the sub-image at the next frame translated by one pixel in every direction. A 3 × 3 matrix is thus generated. The procedure is repeated by translating the sub-image around the highest correlated point as long this point is not in the center of the matrix. The correlation matrix is then interpolated on a 200 × 200 grid, and the maximum of this matrix is taken as the sub-pixel displacement of the cylinder. As the cylinders are aligned with the magnetic field, and do not deform, we do not need to use a rotation or strain correlation, thus saving processing time. We take the maximum of the correlation as the new sub-pixel position of the cylinder. To avoid drift, we keep the same sub-image as reference for subsequent frames, translated to the new pixel position, until the correlation drop below a threshold of 0.9.

#### Determination of the precision on the displacement

The precision of both bead and cylinder displacement is evaluated by following a pair formed by a 4.5 *μ*m magnetic bead and a 6 × 10 *μ*m magnetic cylinder in a magnetic field of 20 mT (Fig. 3a–c). The dipolar force between the bead and the cylinder ensures their close contact, but the pair is subject to Brownian motion. The position of the bead is tracked, for 500 frames, by calculating the center of mass of gray levels in the central spot of beads images focused on the top, while the position of the cylinder is measured by the image correlation algorithm described earlier. The displacement obtained by the two methods give extremely close results (Fig. 3b): the difference between this two position determinations has a standard deviation of 0.045 pixel, or 2.5 nm (Fig. 3c). As the two methods are completely independent, this difference is the quadratic sum of the errors of both methods. Thus both the center determination of beads and the image correlation on cylinders have an error $\le 2.5$ nm.

#### Absolute distance measurements

The cylinder surface position is estimated by a threshold on gray level contrast between the edge of the cylinder and the background with a limited precision due to the diffraction of light. To correct this measurement and evaluate its accuracy, we self-assembled $1\,\mu m$ beads (Dynabeads MyOne) and cylinders in a magnetic field to obtain mixed chain where particles are in contact (see figure Fig. 3B). Although the beads closest to the surface of the cylinder are blurred by diffraction, the third and subsequent beads are perfectly visible and can be localized by using the gray level center of mass of the central spot as before. From this position and the size of the monodispersed beads, the true position of the cylinder surface can be calculated within a few 10’s nm precision thanks to the low polydispersity of dynabeads and compared to the initial estimation. This calibration has been performed on a large number of cylinders, giving an estimation of the systematic error and the standard deviation of the initial estimation. In our illumination system, the simple estimation overvalues the cylinders by 3.9 pixels, with a standard deviation of 2.0 pixels. The systematic error was thus added to our estimation of the distance between two cylinder bases. The error on the *absolute* distance between the two faces (giving the length of the gel or of the cylinder) is the quadratic sum of the estimation error for both faces, or 0.178 *μ*m. This analysis was carried on for both type of cylinder bases (convex and flat coming from respectively top and bottom position in the fabrication wells) and did not show significant differences.

#### Magnetic characterization

For M450 dynabeads, the magnetization curve as a function of the excitation field has been measured by Fonnum *et al*.^20^ and can be used directly. The magnetization curve of the small colloids comprising our cylinders has been measured by Johnson *et al*.^21^. This curve can be used to calculate the magnetization curve for the cylinders using the volume fraction of small colloids in the cylinders. Experimentally cylinders are placed in a field gradient and the velocity of their drift is measured far from the walls (see kymograph on top of Fig. 4A). From this velocity one can easily calculate the viscous force exerted by the surrounding fluid and compare it to the calculated magnetic forces assuming same volumic fraction of the small colloids before and after reticulation of the solution. These forces are in good agreement as can be seen on Fig. 4A confirming that the volumic fraction in the cylinders is similar to the one in the initial solution. This check is done for each new batch of cylinders. From the standard deviation of the ratio of the magnetic force to the viscous force, we can estimate the variability in the magnetic moment between different cylinders. This standard deviation is 7.5%, and by deducing the error from the measurement method we obtain a value of 6.1% on the standard deviation for the magnetization of a cylinder. This small variability is likely due to the variation in small magnetic bead content of the cylinders.

#### Force calculation

We used Comsol Multiphysics to calculate the magnetic dipolar force between two cylinders. The geometry was taken as two coaxial cylinders with rounded edges of 0.5 *μm* radius. The relationship between magnetic flux density and magnetic field strength (B-H curve) is defined for the cylinder based on magnetization curve. The magnetic flux density is fixed at the desired value on two faces of the bounding box. The mesh size was reduced until the results converged. Parametric sweeps were realized for different distances, diameters and cylinder lengths.

In practice, the diameter of the cylinders is fixed by the lithographic mask dimensions and is always the same in a batch. In contrast, the length can vary from one cylinder to the other depending on the filling of the PDMS wells. The length of each cylinder used in the experiments was measured on individual images and for each step of distance between the surfaces, the force at a given excitation field was interpolated from our tabulated forces. In that manner, the relationship between force and distance at a constant excitation field was computed for each pair of specific cylinders used in our experiment. At each image frame, the excitation field and the distance between the cylinders were used to calculate the magnetic force from this relationship.

The main statistical error in the determination of the force is likely the variation in the magnetization of each cylinders. As the force between cylinders is proportional to the magnetization of both of the (independent) magnetizations of the cylinders, we obtain an estimated error of $\sqrt{2}\ast 6.1 \% =\mathrm{8.6 \% }$ on the force between cylinders.

## Discussion

In biophysics, to generate forces and to probe the response of single molecules and cells, three techniques are mainly used: optical trap, atomic force microscopy and magnetic tweezers^22^. Optical traps are limited to forces of 100 pN which are low to probe dense cytoskeletal structures. Atomic force microscope can generate high forces only limited by the stiffness of the cantilever. However to probe actin networks *in vitro*, one has to devote each experiment to one structure grown on the cantilever or a micropattern^11,13^. The experiment presented here is thought as a response to the low throughput of these atomic force microscope experiments: Thanks to the self-organization of the magnetic cylinders, the assembly of the measurement device is rather simple, there is no need to trap a bead or to approach a cantilever. Additionally, one can choose between all chains present in the observation chamber the one that will allow to study multiple gels in parallel. At the end, the combination of these two aspects allows for a very good throughput as compared to other methods.

Magnetic tweezers are a major biophysical technique used to probe single molecules or cellular structures^23^, but the traditional techniques does not allow the generation of high (nN) forces in a large space. In the following, we will explain how we resolve this issue by using homogenous magnetic field. In typical magnetic tweezers experiments, a gradient of magnetic field is created by a permanent- or an electro-magnet. The force exerted on a magnetic particle in such a gradient is $F=m\mathrm{.}\nabla B$, where $m$ is the magnetic moment of the particle and $B$ the magnetic excitation field. In first approximation, for a pair of magnets with a gap of size $d$ or a magnetic tip of smallest dimension $d$ in the core of an electromagnet, a gradient of the order $B/d$ can be obtained at a distance $d$. As the maximum field is capped at 1.3 Tesla for a permanent magnet and 0.5 Tesla for an electromagnet^23^, the main handle to get a steeper gradient and hence a stronger force is the dimension $d$. Hence, to exert nanonewton forces on biological objects such as cells, usual magnetic tweezers setups rely on electromagnets equipped with a sharp tip ($d=10\,\mu m$) that is approached close to the sample by a micromanipulator^24^ or by moving the microscope stage close to the tip^25,26^. In these cases the exerted force is very local (few tens *μ*m from the tip). An homogenous gradient can be obtained on a less local millimeter scale, but here the gradient is less steep and the force is on the order of a few tens of piconewtons^27^. In the technique presented here, we generate an homogenous field with negligible gradient with electromagnets centimeters away from the sample. We use the magnetic dipolar force between the cylinders to generate a force. The dipolar force can be seen as the force exerted on one cylinder by the magnetic gradient generated by the other particle. Hence, the characteristic distance $d$ generating the gradient is the size of the cylinder - a few *μ*m- and nanonewton forces are therefore generated. The location where the force can be generated is not limited to few *μ*m but act on each pair of cylinders in the whole cm-size space between the two coils. With this technique an important limitation of magnetic tweezers is circumvented, as nanonewton forces are generated in a non-localized manner.

Using an homogenous magnetic field also allows for self-organization of cylinders in the whole sample. Several chains are typically assembling, each containing several actin networks (typically from one to five). Monitoring the growth of several chains in parallel is in principle possible by translating the field of view. The variations in the magnetic field and in the resulting mechanical stress will however be experienced by all chains in the experimental chamber as we use a homogenous magnetic field and not a local gradient. If one choose to monitor only one chain, a chain containing the most alternation of functionalized and passivated cylinders can be selected (like the one presented in Fig. 1). It is both the simplicity of the self-organization and the parallel observation of several networks that bestow a good throughput to these experiments.

### Growth speed as a function of stress

The method presented here allows the growth speed of an Arp2/3 actin network to be precisely measured by following the relative displacement of the two cylinders adjoining the network. The two cylinders impose a force on the network that can be varied to apply stresses from a fraction of Pascal to a hundred Pascal. We observed a significant decrease of the growth speed as a function of the applied stress. This observation cannot be due solely to the compression of the network that grew while force was applied: in the time frame necessary to measure the growth speed (≤15 sec), the length of the gel increases by a few percent ($< \mathrm{5 \% }$) when subject to negligible force. Even for a strong elastic deformation of the order of 0.2 at strong stresses, the change in the growth speed would thus only be of the order of 0.01 which is much smaller than the measured decrease of the growth speed. Hence a true modification of the growth of the actin gel is at play.

After a first steep decrease below 10 Pa, the observed decrease of the growth speed is roughly linear up to the maximum measured stress of 100 Pa. This is perfectly compatible with previous measurements on similar biochemical systems^10,13^, but not with previous measurements on actin gels reconstituted from cell extracts^12^ showing a constant velocity up to a strong stress. The range of force we can probe is not sufficient to determine if the growth speed level off, and thus if the behavior is compatible with an exponential decrease at stronger stresses. This limitation could be circumvented by probing growing actin networks that apply force on the colloid with fewer growing ends.

### Non linear mechanics

The elastic modulus we measured is strongly non-linear with a stress-stiffening behavior that has already been reported by others^12,13^. In our previous experiments probing actin networks^14^ we were using spherical colloids on which growth is associated with the build-up of internal stress due to the presence of an elastic spherical shell of actin being pushed outward by the newer polymerizing network. Estimates of this internal stress attain up to 1000 Pa^28^. We compared the elastic modulus of intact spherical shells and shells with a notch denoting symmetry breaking and only found a difference of a factor 2. Our present results on actin networks grown from flat surfaces indicates that symmetry breaking did not release the entire internal stress present in this geometry but merely a fraction of it.

A striking feature of the linear dependance of the elastic modulus on the stress that we observe is that small stresses of the order of 1 Pa corresponds to vanishing elastic modulus on the order of 20 Pa. The fact that this was not observed in previous experiments may not be surprising. As already noted above, internal stresses in a shell of actin are well above this range, forbidding such measurement. On AFM experiments, the minimal stress used during the growth of the network is between 25 Pa^13^ and 100 Pa^12^. In contrast, in our experiments growth is maintained with a stress well below 1 Pa. This is possible because the network is not maintained between the surfaces of two macroscopic objects (glass slide and cantilever) but between the surfaces of microscopic objects (micro-cylinders) subject to Brownian motion. The stress at which the actin network was submitted during its growth is likely to have an effect on its architecture. A crucial architectural feature of actin network grown with Arp2/3 machinery is its branched nature. Each Arp2/3 connects three segments of actin. Ignoring the entanglements, a branched network thus has a very low connectivity compared to networks with more usual crosslinks where each point of contact connects four segments. This connectivity is crucial in determining the mechanical properties of the network^29,30^. Below a critical threshold that can be thought of as a percolation point, the network is floppy and cannot resist deformation. Just above this threshold, the elasticity varies by orders of magnitude as the connectivity is increased^30^. We expect the branched networks studied here, grown at very low force and without crosslinkers to present such a critical state, which would explain the vanishing elastic modulus at low stress. More theoretical development is needed to apply quantitatively these concepts to our system.

## Conclusion

The method we used here to determine the mechanical and growth properties of branched networks can be used to study many other biochemical reconstructions of the cytoskeleton. Of particular interest is the crucial interaction between actin nucleators in the formation of actin networks^31^. Cylinders could be functionalized at the same time with Arp2/3 activators and actin elongators like formin, or tandem monomer binding nucleators to decipher their cooperative work in force production. Other components of the cytoskeleton could also be incorporated in this system. Microtubule asters could be attached to one face of cylinders to probe the mechanics and force generation of these filaments. Microtubules are known to accelerate actin nucleation^32^ in a manner that could be quantified with the method described here. Conversly, intermediate filaments like vimentin also interacts with actin branched network in a way that could be further investigated with this technique^33^. Due to their self organization, magnetic cylinders may also become an object of choice to deform and probe mechanically micron-size biological structures, such as bacteria or decellularized cell nucleus. The advantage of magnetic cylinders over AFM is the simplicity of the experiments and the fact that several structures can be deformed in parallel. Lastly, this technique can be adapted to *in cellulo* measurements, either by using smaller cylinders and inject them into cells to probe intracellular structures, or by using larger magnetic objects to deform whole cells.

## Electronic supplementary material

Supplementary Information
