# Supplementary material for: A new method to measure mechanics and dynamic assembly of branched actin networks
Source: Sci Rep. 2017 Nov 16;7:15688. doi: 10.1038/s41598-017-15638-5 (PMC5691053; doi:10.1038/s41598-017-15638-5)
Supplement: Supplementary file 1 — Supplementary Information [file 41598_2017_15638_MOESM1_ESM.pdf]

A new method to measure mechanics and dynamic assembly of branched actin networks

Pierre Bauer[1], Joseph Tavacoli[1, 2], Thomas Pujol[1, 3], Jessica Planade[1], Olivia du Roure[1,*,+], and Julien Heuvingh[1,*,+]

[1] ESPCI Paris, PSL Research University, CNRS, Universite Pierre et Marie Curie, Universite Paris Diderot, Physique et Mecanique des Milieux Hterogenes, UMR 7636, Paris, 75005, France
[2]Present address: Technical University of Denmark, Department of Energy of Conversion and Storage, Roskilde, Denmark.
[3]Present address: Centre for Genomic Regulation, The Barcelona Institute of Science and Technology, and Universitat Pompeu Fabra, Cell and Developmental Biology, Barcelona, Spain
[*]olivia.duroure@espci.fr, julien.heuvingh@espci.fr

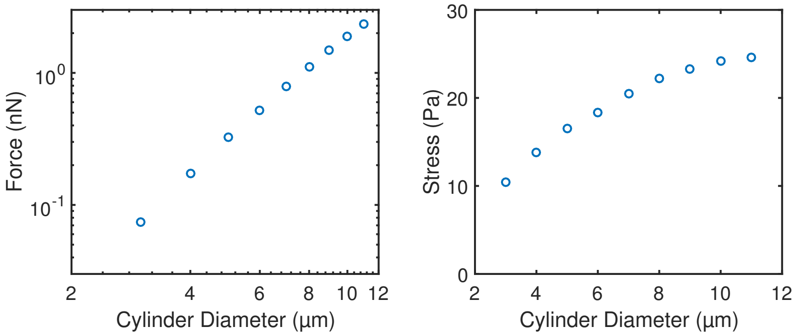

Figure 1: Relationship between force (left) or stress (right) and the diameter of the cylinders calculated by finite element analysis. The cylinder length is fixed at 12 μm, the distance between faces at 2 μm and the external magnetic field at 40 mT

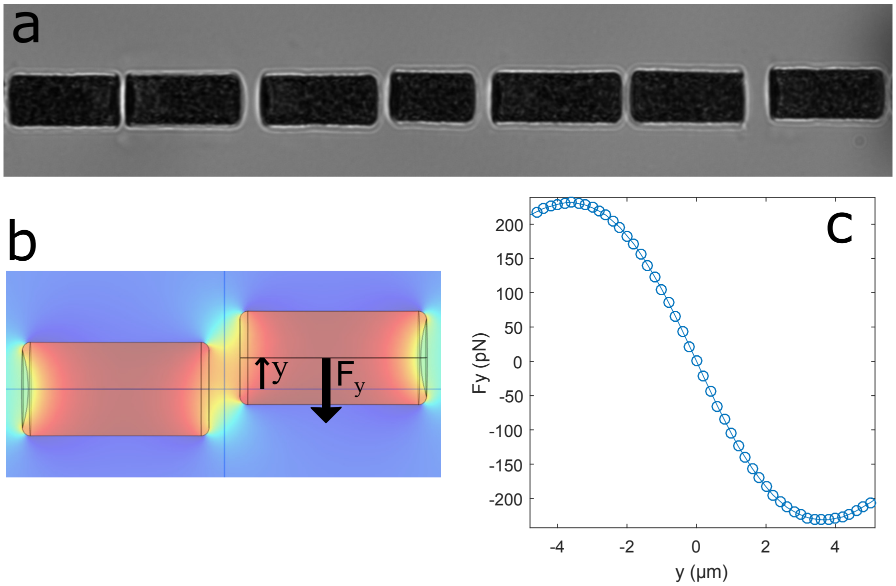

Figure 2: Alignment of magnetic cylinders. a. image of a chain of 6 μm wide cylinders, spaced by a growing actin network. Their is minimal sliding of each cylinder's axis from the chain axis. b. scheme of finite element analysis where one cylinder axis was shifted at a distance y from the axis of the other cylinder. c. Finite element calculation of the transversal force Fy restoring the cylinder's axis to alignment.

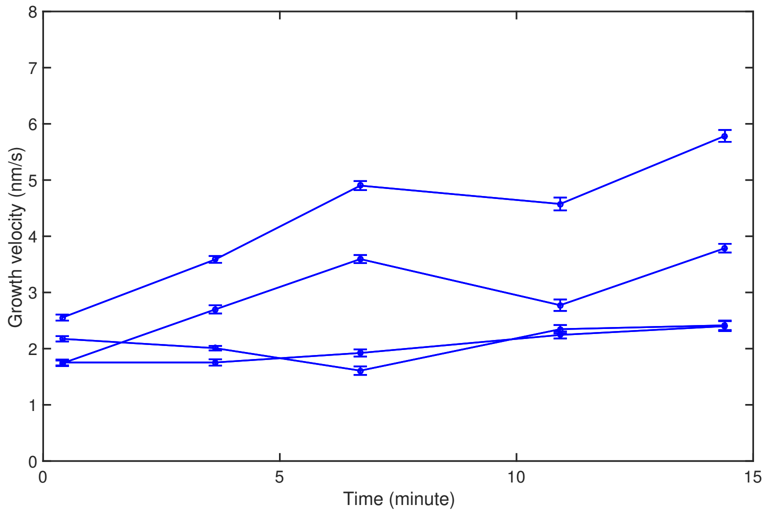

Figure 3: Evolution of the recorded velocities of network growth at stresses below one Pa for four different networks.

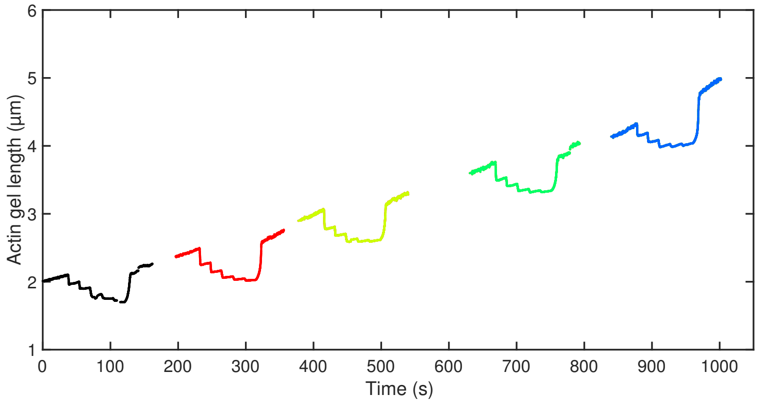

Figure 4: Actin gel length as a function of time while the magnetic field is repeatedly increased: each color represents the actin length when gel is submitted to the stepped increase of the magnetic field from 3 to 80mT. The similarity of the length variation at each repetition suggests an absence of history-dependence.
